# Supplementary material for: A Likelihood Approach for Real-Time Calibration of Stochastic Compartmental Epidemic Models
Source: PLoS Comput Biol. 2017 Jan 17;13(1):e1005257. doi: 10.1371/journal.pcbi.1005257 (PMC5240920; doi:10.1371/journal.pcbi.1005257)
Supplement: S1 File — (TAR.GZ) [file pcbi.1005257.s014.tar.gz › HSPH_Online-SI-Revision/PF08/n1-severe/n1-severe_PF2_table4.pdf]

1	newly 3 weeks spec	24.	14.56	{1., 31.}	54.3333	0.48503	newly 3 weeks cumul	81.	49.01	{9., 90.}	48.9012	0.385174	newly 5 weeks spec	28.	10.89	{0., 22.}	63.6071	0.737918	newly 5 weeks cumul	135.	72.38	{9., 136.}	51.6148	0.443028	newly 50 weeks cumul	286.	141.51	{12., 277.}	56.8077	0.539713
2	newly 3 weeks spec	23.	18.3	{5., 37.}	46.	0.312604	newly 3 weeks cumul	90.	64.65	{24., 115.}	43.1444	0.29609	newly 5 weeks spec	19.	13.44	{2., 26.}	48.1053	0.408775	newly 5 weeks cumul	138.	94.41	{31., 160.}	43.2391	0.310703	newly 52 weeks cumul	204.	163.71	{39., 303.}	48.848	0.334299
3	newly 3 weeks spec	38.	17.02	{3., 33.}	57.6842	0.532279	newly 3 weeks cumul	102.	60.4	{15., 109.}	48.3137	0.374046	newly 5 weeks spec	22.	12.77	{1., 25.}	52.8636	0.538204	newly 5 weeks cumul	152.	89.	{21., 169.}	48.8947	0.389671	newly 51 weeks cumul	261.	168.65	{26., 320.}	50.364	0.427334
4	newly 3 weeks spec	24.	27.26	{10., 49.}	49.4167	0.219876	newly 3 weeks cumul	94.	90.17	{39., 154.}	39.1383	0.192879	newly 5 weeks spec	9.	20.53	{5., 40.}	145.667	0.404417	newly 5 weeks cumul	116.	133.4	{60., 237.}	45.5172	0.188608	newly 54 weeks cumul	163.	253.09	{80., 442.}	77.4785	0.25708
5	newly 3 weeks spec	23.	14.68	{1., 31.}	60.1739	0.571893	newly 3 weeks cumul	56.	52.19	{7., 110.}	62.6607	0.405573	newly 5 weeks spec	17.	10.21	{0., 23.}	61.1176	0.619231	newly 5 weeks cumul	100.	74.7	{11., 168.}	58.06	0.450221	newly 53 weeks cumul	148.	135.33	{12., 322.}	65.7635	0.45938
6	newly 3 weeks spec	43.	16.17	{2., 32.}	62.7209	0.645476	newly 3 weeks cumul	120.	55.27	{8., 109.}	56.425	0.511453	newly 5 weeks spec	35.	12.07	{0., 26.}	66.2	0.756341	newly 5 weeks cumul	192.	80.33	{12., 154.}	59.0573	0.554745	newly 50 weeks cumul	340.	156.91	{12., 296.}	55.2618	0.563071
7	newly 3 weeks spec	17.	13.3	{2., 25.}	54.	0.450873	newly 3 weeks cumul	65.	46.57	{11., 79.}	46.5077	0.359581	newly 5 weeks spec	9.	9.53	{0., 21.}	69.4444	0.474811	newly 5 weeks cumul	90.	67.29	{15., 121.}	47.5667	0.370117	newly 50 weeks cumul	125.	122.82	{19., 300.}	65.52	0.427732
8	newly 3 weeks spec	6.	28.71	{11., 53.}	380.167	0.596162	newly 3 weeks cumul	53.	96.99	{40., 175.}	91.	0.243514	newly 5 weeks spec	0.1	21.05	{6., 41.}	20 952.	2.17023	newly 5 weeks cumul	55.	142.4	{54., 258.}	161.709	0.355958	newly 54 weeks cumul	55.	240.77	{76., 427.}	339.327	0.551658
9	newly 3 weeks spec	21.	12.4	{2., 21.}	54.	0.475682	newly 3 weeks cumul	83.	44.76	{11., 87.}	53.5663	0.444016	newly 5 weeks spec	22.	9.37	{0., 22.}	62.6818	0.743918	newly 5 weeks cumul	119.	64.92	{12., 126.}	53.479	0.463211	newly 50 weeks cumul	236.	120.58	{13., 282.}	60.822	0.576897
10	newly 3 weeks spec	16.	14.53	{2., 26.}	49.6875	0.341281	newly 3 weeks cumul	74.	51.7	{14., 96.}	44.027	0.312813	newly 5 weeks spec	16.	10.2	{2., 20.}	55.875	0.465163	newly 5 weeks cumul	103.	73.96	{19., 140.}	45.2039	0.320998	newly 50 weeks cumul	137.	134.12	{25., 283.}	59.2409	0.349602
11	newly 3 weeks spec	26.	8.72	{0., 19.}	68.	0.818231	newly 3 weeks cumul	70.	31.89	{1., 69.}	57.8143	0.676866	newly 5 weeks spec	28.	7.03	{0., 16.}	74.8929	1.00333	newly 5 weeks cumul	115.	46.95	{1., 102.}	60.3043	0.740786	newly 47 weeks cumul	274.	92.71	{1., 213.}	66.792	0.902719
12	newly 3 weeks spec	14.	16.5	{2., 30.}	61.1429	0.324864	newly 3 weeks cumul	54.	58.65	{19., 97.}	50.0926	0.242072	newly 5 weeks spec	7.	11.5	{2., 24.}	101.429	0.3844	newly 5 weeks cumul	71.	83.08	{28., 151.}	55.1549	0.253168	newly 55 weeks cumul	74.	138.37	{28., 298.}	114.23	0.34859
13	newly 3 weeks spec	28.	15.56	{4., 27.}	51.5714	0.450726	newly 3 weeks cumul	97.	53.19	{16., 90.}	49.8454	0.396063	newly 5 weeks spec	15.	11.24	{1., 22.}	55.3333	0.464217	newly 5 weeks cumul	134.	77.57	{20., 136.}	48.5299	0.388064	newly 50 weeks cumul	214.	147.84	{23., 306.}	51.1402	0.406393
14	newly 3 weeks spec	29.	22.45	{4., 50.}	59.069	0.428354	newly 3 weeks cumul	89.	79.2	{19., 164.}	54.7865	0.32145	newly 5 weeks spec	15.	14.66	{1., 33.}	67.2	0.439818	newly 5 weeks cumul	126.	111.85	{27., 234.}	55.9921	0.337219	newly 55 weeks cumul	170.	179.98	{36., 389.}	64.5765	0.347988
15	newly 3 weeks spec	13.	12.66	{1., 27.}	60.	0.441966	newly 3 weeks cumul	54.	43.75	{6., 84.}	49.2037	0.390818	newly 5 weeks spec	3.	9.55	{0., 21.}	255.667	0.634753	newly 5 weeks cumul	64.	63.6	{6., 119.}	56.1563	0.379247	newly 49 weeks cumul	74.	116.55	{6., 235.}	104.635	0.468238
16	newly 3 weeks spec	18.	18.17	{1., 44.}	70.6111	0.490224	newly 3 weeks cumul	97.	62.86	{6., 133.}	57.0515	0.49557	newly 5 weeks spec	13.	12.54	{0., 30.}	73.0769	0.586142	newly 5 weeks cumul	125.	90.66	{9., 203.}	58.112	0.482731	newly 55 weeks cumul	201.	165.92	{10., 386.}	62.8358	0.511316
17	newly 3 weeks spec	13.	17.99	{2., 37.}	86.8462	0.424688	newly 3 weeks cumul	54.	66.46	{8., 139.}	69.2222	0.346129	newly 5 weeks spec	4.	12.94	{0., 30.}	260.	0.636824	newly 5 weeks cumul	73.	94.43	{11., 196.}	76.7808	0.370176	newly 55 weeks cumul	87.	148.9	{12., 346.}	113.103	0.430647
18	newly 3 weeks spec	19.	15.13	{0., 32.}	60.2632	0.541744	newly 3 weeks cumul	61.	53.55	{5., 106.}	57.5574	0.433779	newly 5 weeks spec	28.	12.68	{0., 28.}	63.8571	0.750823	newly 5 weeks cumul	119.	79.97	{7., 151.}	54.4286	0.499565	newly 55 weeks cumul	388.	164.69	{8., 352.}	65.8892	0.730439
19	newly 3 weeks spec	15.	9.95	{1., 20.}	52.3333	0.447544	newly 3 weeks cumul	71.	36.85	{7., 74.}	55.7042	0.4532	newly 5 weeks spec	12.	7.25	{0., 15.}	57.75	0.589377	newly 5 weeks cumul	93.	52.13	{8., 101.}	53.8817	0.438792	newly 48 weeks cumul	186.	88.4	{10., 208.}	59.5484	0.54769
20	newly 3 weeks spec	7.	10.76	{1., 20.}	94.8571	0.420381	newly 3 weeks cumul	54.	39.34	{8., 72.}	46.2963	0.366386	newly 5 weeks spec	3.	7.88	{0., 17.}	201.333	0.576246	newly 5 weeks cumul	64.	56.74	{8., 102.}	51.1875	0.357825	newly 48 weeks cumul	97.	100.3	{12., 223.}	66.3711	0.41063
21	newly 3 weeks spec	33.	15.45	{0., 30.}	59.9697	0.692721	newly 3 weeks cumul	107.	56.12	{4., 114.}	56.8224	0.55848	newly 5 weeks spec	12.	10.39	{0., 22.}	65.5833	0.599772	newly 5 weeks cumul	135.	79.77	{5., 163.}	54.9556	0.536394	newly 55 weeks cumul	223.	143.81	{5., 308.}	57.0269	0.567322
22	newly 3 weeks spec	12.	13.19	{2., 25.}	59.0833	0.328657	newly 3 weeks cumul	57.	47.3	{11., 88.}	43.6842	0.305779	newly 5 weeks spec	7.	8.46	{2., 18.}	73.7143	0.385034	newly 5 weeks cumul	75.	66.65	{16., 121.}	45.9867	0.306436	newly 49 weeks cumul	81.	114.7	{21., 250.}	81.679	0.349487
23	newly 3 weeks spec	21.	14.7	{4., 30.}	50.	0.345363	newly 3 weeks cumul	88.	49.7	{19., 93.}	49.6591	0.353621	newly 5 weeks spec	16.	12.78	{2., 29.}	56.625	0.421028	newly 5 weeks cumul	122.	76.11	{27., 153.}	48.6311	0.337428	newly 51 weeks cumul	188.	173.41	{40., 361.}	61.0585	0.352495
24	newly 3 weeks spec	15.	11.5	{1., 27.}	60.1333	0.466502	newly 3 weeks cumul	68.	38.06	{10., 84.}	54.5294	0.41573	newly 5 weeks spec	6.	8.28	{0., 21.}	105.333	0.559987	newly 5 weeks cumul	79.	55.89	{12., 122.}	54.8987	0.380775	newly 48 weeks cumul	103.	124.17	{18., 311.}	83.0194	0.391778
25	newly 3 weeks spec	9.	10.38	{1., 22.}	73.3333	0.420667	newly 3 weeks cumul	59.	36.59	{7., 73.}	50.7288	0.433096	newly 5 weeks spec	11.	7.69	{0., 15.}	60.4545	0.583468	newly 5 weeks cumul	83.	53.15	{8., 105.}	51.9398	0.43495	newly 46 weeks cumul	100.	95.23	{10., 194.}	64.33	0.428771
26	newly 3 weeks spec	16.	13.4	{0., 36.}	71.5	0.610203	newly 3 weeks cumul	56.	47.72	{4., 110.}	68.6071	0.509208	newly 5 weeks spec	15.	8.67	{0., 22.}	65.9333	0.745442	newly 5 weeks cumul	84.	67.	{4., 171.}	67.0476	0.534557	newly 53 weeks cumul	169.	111.63	{4., 271.}	64.5148	0.621416
27	newly 3 weeks spec	8.	20.74	{4., 37.}	177.	0.410466	newly 3 weeks cumul	28.	70.03	{21., 130.}	158.607	0.362116	newly 5 weeks spec	0.1	16.1	{3., 33.}	16 008.	2.00789	newly 5 weeks cumul	28.	104.54	{28., 189.}	276.429	0.494907	newly 52 weeks cumul	28.	195.31	{34., 398.}	599.321	0.711516
28	newly 3 weeks spec	36.	20.55	{2., 45.}	56.9167	0.539756	newly 3 weeks cumul	124.	72.22	{8., 158.}	54.7097	0.46587	newly 5 weeks spec	31.	14.91	{1., 35.}	60.7419	0.632914	newly 5 weeks cumul	183.	104.97	{14., 232.}	55.2732	0.47048	newly 55 weeks cumul	256.	194.17	{24., 391.}	54.0977	0.411461
29	newly 3 weeks spec	15.	10.09	{0., 21.}	52.0667	0.508347	newly 3 weeks cumul	52.	36.19	{5., 64.}	44.3269	0.390534	newly 5 weeks spec	10.	8.34	{0., 19.}	67.8	0.572634	newly 5 weeks cumul	71.	53.84	{6., 103.}	47.3239	0.40267	newly 48 weeks cumul	150.	105.58	{7., 247.}	59.1333	0.534165
30	newly 3 weeks spec	15.	12.38	{1., 25.}	60.5333	0.508488	newly 3 weeks cumul	70.	43.82	{4., 87.}	53.2	0.44895	newly 5 weeks spec	2.	9.09	{0., 21.}	389.5	0.710093	newly 5 weeks cumul	78.	63.85	{5., 132.}	56.4487	0.421981	newly 49 weeks cumul	112.	106.77	{6., 263.}	71.8839	0.477264
31	newly 3 weeks spec	19.	13.79	{0., 29.}	57.8421	0.554314	newly 3 weeks cumul	55.	45.77	{5., 87.}	54.5273	0.429256	newly 5 weeks spec	24.	9.91	{0., 23.}	64.375	0.826264	newly 5 weeks cumul	98.	67.72	{6., 137.}	53.8367	0.492291	newly 50 weeks cumul	218.	133.17	{6., 327.}	60.9037	0.637339
32	newly 3 weeks spec	37.	15.64	{1., 32.}	60.3243	0.64316	newly 3 weeks cumul	98.	55.15	{11., 103.}	51.051	0.419806	newly 5 weeks spec	27.	11.85	{0., 26.}	61.8889	0.725997	newly 5 weeks cumul	151.	80.55	{14., 150.}	52.7219	0.460182	newly 52 weeks cumul	280.	139.61	{14., 306.}	57.5107	0.555719
33	newly 3 weeks spec	31.	18.87	{2., 45.}	59.1935	0.560644	newly 3 weeks cumul	119.	69.89	{11., 138.}	55.437	0.515381	newly 5 weeks spec	13.	12.48	{1., 28.}	65.5385	0.484993	newly 5 weeks cumul	150.	97.2	{13., 213.}	53.8133	0.498855	newly 55 weeks cumul	184.	145.64	{15., 318.}	55.9239	0.478189
34	newly 3 weeks spec	15.	9.7	{1., 20.}	56.9333	0.519583	newly 3 weeks cumul	64.	33.98	{5., 70.}	54.7188	0.470388	newly 5 weeks spec	10.	7.16	{0., 18.}	63.	0.564601	newly 5 weeks cumul	87.	48.94	{8., 101.}	53.8161	0.463528	newly 47 weeks cumul	179.	95.9	{8., 245.}	61.0279	0.579921
35	newly 3 weeks spec	21.	21.1	{4., 44.}	50.4762	0.303817	newly 3 weeks cumul	87.	76.04	{21., 135.}	41.1494	0.278901	newly 5 weeks spec	3.	14.48	{2., 31.}	398.	0.641298	newly 5 weeks cumul	98.	107.41	{30., 194.}	51.1122	0.280736	newly 55 weeks cumul	98.	172.96	{45., 318.}	99.8163	0.342325
36	newly 3 weeks spec	1.	14.3	{2., 29.}	1334.	0.995089	newly 3 weeks cumul	11.	54.52	{15., 110.}	398.364	0.600646	newly 5 weeks spec	0.1	9.75	{0., 23.}	9676.	1.62325	newly 5 weeks cumul	11.	75.92	{19., 156.}	591.818	0.719641	newly 56 weeks cumul	11.	123.6	{22., 268.}	1024.73	0.870474
37	newly 3 weeks spec	19.	8.67	{1., 18.}	59.3158	0.627131	newly 3 weeks cumul	60.	30.37	{5., 64.}	54.8167	0.487308	newly 5 weeks spec	11.	6.33	{0., 16.}	61.	0.662298	newly 5 weeks cumul	82.	44.42	{7., 90.}	54.122	0.482004	newly 46 weeks cumul	171.	80.73	{8., 185.}	62.4503	0.61986
38	newly 3 weeks spec	5.	21.62	{8., 40.}	337.2	0.576452	newly 3 weeks cumul	41.	73.59	{32., 136.}	90.122	0.250627	newly 5 weeks spec	3.	15.57	{5., 31.}	423.667	0.624439	newly 5 weeks cumul	46.	107.3	{47., 199.}	139.174	0.333771	newly 54 weeks cumul	51.	206.54	{60., 420.}	309.608	0.5103
39	newly 3 weeks spec	20.	12.99	{1., 24.}	49.05	0.450168	newly 3 weeks cumul	64.	44.37	{9., 77.}	44.4844	0.354422	newly 5 weeks spec	14.	9.56	{0., 22.}	58.7143	0.559582	newly 5 weeks cumul	91.	66.01	{9., 120.}	45.8352	0.371591	newly 50 weeks cumul	151.	125.5	{9., 302.}	58.596	0.450952
40	newly 3 weeks spec	21.	15.8	{1., 33.}	59.9048	0.506297	newly 3 weeks cumul	63.	58.65	{7., 114.}	58.0159	0.376028	newly 5 weeks spec	9.	11.32	{0., 23.}	92.6667	0.567795	newly 5 weeks cumul	89.	83.59	{8., 167.}	59.0225	0.388509	newly 56 weeks cumul	118.	135.17	{8., 279.}	71.9915	0.420391
41	newly 3 weeks spec	14.	23.38	{7., 50.}	93.8571	0.279533	newly 3 weeks cumul	52.	82.17	{30., 150.}	80.4423	0.242334	newly 5 weeks spec	6.	15.83	{2., 35.}	187.5	0.429316	newly 5 weeks cumul	63.	117.71	{39., 227.}	103.54	0.270195	newly 53 weeks cumul	66.	194.09	{54., 410.}	202.742	0.382922
42	newly 3 weeks spec	25.	12.67	{0., 31.}	65.	0.752186	newly 3 weeks cumul	82.	43.57	{2., 106.}	62.3293	0.650937	newly 5 weeks spec	15.	8.88	{0., 21.}	67.0667	0.765029	newly 5 weeks cumul	118.	62.65	{2., 162.}	63.3814	0.674564	newly 53 weeks cumul	257.	114.1	{2., 271.}	66.1167	0.791233
43	newly 3 weeks spec	9.	8.13	{0., 22.}	79.	0.638246	newly 3 weeks cumul	28.	28.77	{1., 66.}	71.6786	0.498363	newly 5 weeks spec	9.	5.99	{0., 13.}	68.5556	0.745619	newly 5 weeks cumul	44.	41.56	{2., 105.}	69.9545	0.540712	newly 46 weeks cumul	120.	79.32	{3., 229.}	69.3	0.70817
44	newly 3 weeks spec	16.	17.36	{1., 39.}	75.25	0.50491	newly 3 weeks cumul	64.	61.57	{6., 143.}	64.2031	0.408966	newly 5 weeks spec	5.	11.97	{0., 29.}	189.8	0.625632	newly 5 weeks cumul	85.	87.13	{9., 201.}	68.5529	0.41476	newly 54 weeks cumul	109.	150.86	{15., 327.}	94.1284	0.447626
45	newly 3 weeks spec	17.	16.8	{5., 32.}	50.7059	0.271471	newly 3 weeks cumul	55.	56.44	{24., 108.}	42.0727	0.191383	newly 5 weeks spec	8.	13.96	{2., 27.}	109.75	0.39064	newly 5 weeks cumul	80.	85.59	{28., 157.}	46.2625	0.206715	newly 51 weeks cumul	157.	180.95	{46., 369.}	65.9299	0.290724
46	newly 3 weeks spec	36.	13.75	{2., 30.}	62.8056	0.624077	newly 3 weeks cumul	87.	47.29	{11., 85.}	50.2414	0.393478	newly 5 weeks spec	13.	10.69	{1., 21.}	56.8462	0.451651	newly 5 weeks cumul	135.	69.98	{17., 127.}	51.3926	0.42008	newly 50 weeks cumul	318.	133.77	{24., 286.}	58.7075	0.56204
47	newly 3 weeks spec	0.1	29.18	{8., 48.}	29 080.	2.36327	newly 3 weeks cumul	20.	103.71	{41., 165.}	419.55	0.659062	newly 5 weeks spec	0.1	18.25	{3., 33.}	18 158.	2.08432	newly 5 weeks cumul	20.	145.87	{51., 245.}	629.75	0.794993	newly 56 weeks cumul	20.	221.88	{60., 365.}	1009.6	0.954124
48	newly 3 weeks spec	16.	16.59	{4., 33.}	55.6875	0.307938	newly 3 weeks cumul	39.	56.96	{17., 104.}	71.641	0.251987	newly 5 weeks spec	12.	13.29	{1., 27.}	71.25	0.417869	newly 5 weeks cumul	57.	86.22	{22., 155.}	77.5088	0.269057	newly 50 weeks cumul	102.	176.28	{33., 345.}	105.961	0.34519
49	newly 3 weeks spec	13.	14.61	{2., 34.}	72.2308	0.403286	newly 3 weeks cumul	64.	53.78	{12., 107.}	56.4063	0.402423	newly 5 weeks spec	9.	9.96	{1., 22.}	77.1111	0.462084	newly 5 weeks cumul	90.	74.34	{15., 155.}	57.5333	0.421753	newly 54 weeks cumul	127.	119.39	{17., 315.}	67.4252	0.451876
50	newly 3 weeks spec	21.	16.92	{3., 30.}	47.2381	0.363935	newly 3 weeks cumul	56.	58.43	{14., 100.}	49.9464	0.269529	newly 5 weeks spec	17.	12.19	{0., 24.}	55.3529	0.533727	newly 5 weeks cumul	91.	85.47	{16., 146.}	47.7912	0.296173	newly 52 weeks cumul	172.	156.47	{16., 320.}	55.843	0.382486
